# The Impact of X-Chromosome Inactivation on Phenotypic Expression of X-Linked Neurodevelopmental Disorders

**DOI:** 10.3390/brainsci11070904

**Published:** 2021-07-09

**Authors:** Boudewien A Brand, Alyssa E Blesson, Constance L. Smith-Hicks

**Affiliations:** 1 Center for Autism and Related Disorders, Kennedy Krieger Institute, Baltimore, MD 21205, USA; bobrand13@gmail.com (B.A.B.); Blesson@kennedykrieger.org (A.E.B.); 2 Department of Neurology and Developmental Medicine, Kennedy Krieger Institute, Baltimore, MD 21205, USA; 3 Department of Neurology, Johns Hopkins University School of Medicine, Baltimore, MD 21287, USA

**Keywords:** X-chromosome inactivation, MECP2, FMR1, Rett syndrome, fragile X syndrome, FXTAS, POI, neurodevelopmental disorders

## Abstract

Nearly 20% of genes located on the X chromosome are associated with neurodevelopmental disorders (NDD) due to their expression and role in brain functioning. Given their location, several of these genes are either subject to or can escape X-chromosome inactivation (XCI). The degree to which genes are subject to XCI can influence the NDD phenotype between males and females. We provide a general review of X-linked NDD genes in the context of XCI and detailed discussion of the sex-based differences related to *MECP2* and *FMR1*, two common X-linked causes of NDD that are subject to XCI. Understanding the effects of XCI on phenotypic expression of NDD genes may guide the development of stratification biomarkers in X-linked disorders.

## 1. Introduction

Several genes on the X chromosome are specifically expressed in the brain and are essential for neuronal plasticity and cognitive processes [1]. Of these, nearly 20% have been linked to neurodevelopmental disorders (NDD) and the dissimilar phenotype in males and females is due in part to differences in the pattern of gene expression [2]. The hemizygosity of most X-linked genes reveals recessive phenotypes in males, thus accounting for the disproportionately large number of affected males [3], while females with the same pathogenic variant are often unaffected or mildly affected. The sex differences found in X-linked NDDs are influenced by X-chromosome inactivation (XCI), a method of X-chromosome dosage compensation that ensures that X-linked genes are expressed at the same level in females as in males. Very early in female development, random inactivation of either the paternal or the maternal X chromosomes occurs in each cell, and the pattern of inactivation is transmitted to all daughter cells via mitosis. This results in the mosaic expression of X-linked genes in females, which can confer protection against disease. Generally, the ratio of the expression of maternal and paternal alleles is about 50:50 in females; however, deviation from the 50:50 ratio, known as skewed XCI, is also seen. Skewed XCI occurs when the inactivation of one X chromosome is favored over the other, and the ratio is commonly considered skewed if it is ≥65:35.

In this review we provide a general overview of X-linked NDD genes, their phenotype and association with XCI, and a focused discussion of the phenotypes associated with methyl-CpG binding protein 2 (*MECP2*) and fragile X mental retardation 1 (*FMR1*), two genes that are subject to XCI.

## 2. X-Linked NDD Genes

The majority of X-linked NDD genes are subject to XCI, resulting in phenotypic variability between males and females. In Table 1, we show a representative sample of genes that have a well-established association with an NDD phenotype as notated in OMIM [4] and discussed in the review by Migeon [5]. Several factors influence clinical presentation in females; these include whether the gene escapes XCI or is subject to skewing, the variant type, as well as the inheritance pattern. About 15% of the genes on the X chromosome escape inactivation and are expressed from both the active and inactive chromosomes [4]. The degree of the escape from XCI is reported to vary between genes, tissues, and individuals and likely contributes to phenotypic heterogeneity [6,7]. For genes that escape XCI, the NDD phenotype may be lethal in males and is generally more severe when compared with symptomatic females. This pattern is seen in X-linked disorders associated with the following genes: *SMC1A*, *USP9X*, *LAMP2*, *IQSEC2*, *DCX*, *DDX3X*, and *OFD1* (Table 1).

Of the genes that are subject to XCI, skewing towards expression of the allele with the pathogenic variant or the normal allele may occur. When skewing is towards the variant allele, females and males show a similar affected phenotype, as seen in WDR45-related disorders. Conversely, when there is skewing towards expression of the normal allele, females are typically asymptomatic. An exception to this pattern is seen with the *ABCD1* gene, where there can be a less severe phenotype in females, despite skewing towards expression of the variant allele [8].

**Table 1:** Impact of XCI on NDD.

Gene	X-Linked Disorder	Inheritance	Male	Female	Gene Subject to X-Inactivation	References
*ABCD1*	Adrenoleukodystrophy/Adrenomyeloneuropathy	Recessive	Death first decade/progressive stiffness and weakness in the legs, development of cognitive and behavioral disturbance beginning in the 2nd decade	unaffected/late onset adrenomyeloneuropathy	Yes	PMID: 23469258 [9]; PMID: 22280810 [10]
AFF2	Intellectual developmental disorder, X-linked 109	Recessive	Global developmental delay/ID/behavioral dx	mild or unaffected	N.D.	n/a
AIFM1	Spondyloepimetaphyseal dysplasia, X-linked, with hypomyelinating leukodystrophy	Recessive	hypomyelinating leukodystrophy	unaffected carrier females	Yes	PMID: 32337346 [11]
ALG13	Developmental and epileptic encephalopathy 36		early-onset epileptic encephalopathy, severe intellectual disability	developmental and epileptic encephalopathy-36; unaffected carrier females	Yes	PMID: 28778787 [12]
AP1S2	PETTIGREW SYNDROME	Recessive	Intellectual disability	unaffected carrier females	ND	n/a
ARHGEF9	Developmental and epileptic encephalopathy 8	Recessive	profound ID, epilepsy	intellectual disability, unaffected carriers	Yes	PMID: 33600053 [13]
*ARSE*	Chondrodysplasia punctata 1	Recessive	Developmental delay/ID	not described	ND	n/a
*ARX*	Early infantile epileptic encephalopathyLISX2, Proud syndrome	Recessive	epilepsy and profound ID; brain abnormalities, abnormal genitalia	unaffected; mild phenotype	Yes	PMID: 21416597 [14]
ATP6AP2	Intellectual Disability, X-linked, syndromic, Hedera type	Recessive	ID, epilepsy parkinsonism, spasticity	unaffected	N.D.	n/a
ATP7A	Menkes disease	Recessive	epilepsy, developmental delay	unaffected	N.D.	n/a
*ATRX*	Alpha-thalassemia/ID	Dominant	Severe ID and dysmorphic features	mild ID	Yes	PMID: 16100724 [15]
*ATRX*	ATRX ID syndrome	Recessive	severe ID and dysmorphic features	unaffected	Yes	PMID: 16955409 [16]
BRWD3	Intellectual Disability, X-linked 93	Recessive	ID	unaffected carrier females	N.D.	n/a
CASK	Intellectual Disability and microcephaly with pontine and cerebellar hypoplasia	Dominant	ID, microcephaly, pontine, cerebellar hypoplasia	unaffected carrier females, ASD	Yes	PMID: 28944139 [17]
*CDKL5*	Early infantile epileptic encephalopathy early death	Dominant	milder phenotype, epilepsy and profound ID	severe ID, early onset epilepsy, microcephaly, less severe	Yes	PMID: 24564546 [18]
CLIC2	Intellectual Disability, X-linked, syndromic 32	Recessive	no affected males	mild learning disabilities	N.D.	n/a
*CLCN4*	Raynaud-Claes syndrome	Dominant	Sever ID and epilepsy	milder phenotype	Yes	PMID: 27550844 [19]
*CNKSR2*	Houge type	ND	epilepsy, microcephaly, developmental delay	mild ID, seizure	Yes	PMID: 31414730 [20]
*CUL4B*	Cullun Ring Cabezas type	Recessive	syndromic ID	learning disability	Yes	PMID: 17273978 [21]
*CXorf56*	CXorf56-Associated ID	ND	moderate ID	generally unaffected or mild phenotype	Yes	PMID: 31822863 [22]
*DCX*	Lissencephaly	ND	ID, epilepsy, brain malformation	mild epilepsy	Yes	PMID: 12838518 [23]
*DDX3X*	Snijders Blok	Recessive	some males with non-syndromic ID	ID, microcephaly	escapes X inactivation	PMID: 30871455 [24]
*DLG3*	Intellectual Disability, X-linked 90	Recessive	moderate - severe ID	not affected and affected	Yes	PMID: 28777483 [25]
*DMD*	Duchenne, Muscular dystrophy	Recessive	mild ID	unaffected	Yes	PMID: 27098336 [26]
FAM50A	Intellectual developmental disorder, X-linked, syndromic, Armfield type		ID	unaffected	N.D.	n/a
*FDG1*	Aarskog–Scott syndrome	Not reported	Facio-genetial dysmorphisms, ADHD, ID	short stature	N.D.	n/a
*FGF13*	Developmental and epileptic encephalopathy 90	Dominant/Recessive	epilepsy, developmental delay	epilepsy, developmental delay	N.D.	n/a
*FMR1*	Fragile X syndrome	Dominant	ID	mild	Yes	PMID: 8825916 [27]
*FMR1*	Fragile X Tremor Ataxia	Dominant	late onset tremor, ataxia, cognitive decline	FXTAS in 10% of premutation carriers	Yes	PMID: 26609701 [28]
*FMR1*	Premature Ovarian Failure		n/a	POI in 25% of premutation carriers	N.D.	PMID: 30098699 [29]
FRMPD4	Intellectual Disability, X-linked 104		ID	unaffected	N.D.	n/a
FTSJ1	Intellectual Disability, X-linked 9/44	Recessive	ID and mood disorder	unaffected carrier females	N.D.	n/a
GRIA3	Intellectual developmental disorder, X-linked, syndromic, Wu type	Recessive	ID	unaffected carrier females	Yes	PMID: 19449417 [30]
*GPC3/GPC4*	Simpson-Golabi-Behmel	Recessive	ID, congenital malformation	generally unaffected	Yes	PMID: 30048822 [31]
*HCFC1*	Methylmalonic acidemia	Recessive	ID	not affected	N.D.	n/a
*HDAC8*	Cornelia de Lange, 5	Dominant	syndromic ID	mild	Yes	PMID: 22889856 [32]
*HPRT*	Lesch-Nyhan syndrome	Recessive	ID, spastic cerebral palsy and SIB	not affected	Yes	PMID: 6585829 [33]
*HUWE1*	Intellectual Disability, X-linked	Not reported	moderate -profound syndromic ID	Chiari malformation, ID, dysmorphism	N.D.	n/a
IGBP1	Corpus callosum, agenesis of, with Intellectual Disability, ocular coloboma and micrognathia	Recessive	ID	unaffected	N.D.	n/a
IL1RAPL1	Intellectual Disability, X-linked 21/34	Recessive	ID, microcephaly	unaffected carrier females	N.D.	n/a
*IQSEC2*	Intellectual Disability, X-linked 1/78	Dominant	non-syndromic ID, epilepsy and non-syndromic ID	some with learning disability, milder ID some with epilepsy	escapes X inactivation	PMID: 32564198 [34]
*KDM5C/JARIDC/SMCX*	Claes-Jensen	Recessive	microcephaly, developmental disability	mild phenotype	Yes	[35]
*KDM6A (UTX)*	Kabuki syndrome 2	Dominant	syndromic ID	similar to males	escapes X inactivation	PMID: 29022598 [7]
KLHL15	Intellectual Disability, X-linked 103	Recessive	ID, epilepsy, brain malformation	mild or unaffected	Yes	PMID: 24817631 [36]
*L1CAM*	Hydrocephalus. X-linked aqueductal stenosis	Recessive	ID, spastic paraplegia	mild ID, some are not affected	N.D.	n/a
*LAMP2*	Danon disease	Dominant	ID and myopathy	late onset	escapes X inactivation	PMID: 30871455 [24]
*MAOA*	Monoamine oxidase A def	Recessive	mild ID, behavioral difficulties	not affected	Yes	PMID: 19684479 [37]
*MECP2*	Rett syndrome	Dominant	early infantile epileptic encephalopathy/death first 2-4 yrs of life	ID, epilepsy, microcephaly, gait and language disorder	Yes	PMID: 18361425 [38]; PMID: 31427717 [39]
*MECP2*	MECP2 Dup Syndrome	Recessive	profound Intellectual Disability, infantile hypotonia, autistic features, seizures, progressive spasticity, and recurrent infections	mild neuropsychiatric features, such as anxiety.	Yes	PMID: 29141583 [40]
*MECP2*	PPMX	Recessive	ID, spasticity, tremor, hyperkinetic behavior	unaffected carrier females	N.D.	n/a
*MED12 (HOPA)*	MED12-Related Disorders	Recessive	moderate ID, marfanoid habitus, ID, ptosis, cryptorchidism	unaffected carrier females or Hardikar syndrome	Yes	PMID: 33244166 [41]
*NEXMIF (KIA2022)*	neurite extension & migration	Dominant	severe ID, epilepsy	unaffected, intractable epilepsy and ID	Yes	PMID: 27358180 [42]; PMID: 29717186 [43]
*NHS*	Nance–Horan Syndrome	Dominant	Congenital cataract, microphthalmia, and mild or moderate	mild vision impairment	N.D.	n/a
NKAP	Intellectual developmental disorder, X-linked, syndromic, Hackman-Di Donato type	Recessive	ID	unaffected carrier females	N.D.	n/a
NLGN3	Autism risk		ASD	unaffected carrier females	Yes	PMID: 18361425 [38]
NLGN4X	Intellectual Disability, X-linked		no affected males	ID, epilepsy and language disorder	Yes	PMID: 32564284 [44]
NONO	Intellectual Disability, X-linked, syndromic 34		ID, congenital cardiac malformation	unaffected carrier females	N.D.	n/a
*NSDHL*	CK syndrome	Recessive	ID, neonatal seizures		N.D.	n/a
*OCRL1*	Lowe syndrome	Recessive	ID, cataracts	not affected	skewed X-inactivation	PMID: 7180850 [45]
*OFD1*	Simpson-Golabi-Behmel	Recessive	early lethality, severe ID	not affected	escapes X inactivation	PMID: 31243241 [46]
*OFD1*	Joubert 10	Recessive	ID, congenital malformation	unaffected carrier females	N.D.	n/a
*OGT*	Intellectual Disability, X-linked	Recessive	syndromic ID	not affected	random X-inactivation	PMID: 25136351 [47]
*OPHN1*	ID, X-linked Congenital Cerebellar hypoplasia	Recessive	ID, hypotonia, ataxia, seizures, macrocephaly, strabismus, dismorphic features	not reported; mild ID, dysmprohic features, strabismus	Yes	PMID: 24105372 [48]
PAK3	Intellectual Disability, X-linked 30/47	Recessive	ID	unaffected carrier females	N.D.	n/a
*PCDH19*	Early infantile epileptic encephalopathy, 9	Not reported	Mosaic males	ID, autism, infantile seizures	Yes	PMID: 22091964 [49]
*PDHA1*	PDC deficiency	Dominant	early lethality, brain malformation, infantile/childhood onset Leigh mild ataxia	dysmorphism, brain malformation, epilepsy, spastic cp	Yes	PMID: 31673819 [50]
*PHF6*	Borjeson–Forssman–Lehmann syndrome	Recessive	ID, epilepsy	mild ID	Yes	PMID: 15994862 [51], PMID: 12415272 [52], PMID: 22190899 [53]
*PHF8*	Syndromic X-linked intellectual disability Siderius type	Recessive	syndromic ID	not affected	Yes	PMID: 18498374 [54]
*PLP1*	Pelizaeus-Merzbacher Disease	Recessive	leukodystrophy and spastic diplegia	mild or unaffected	Yes	PMID: 10878666 [55]; PMID: 12297985 [56]
*PORCN*	Focal dermal hypoplasia	Dominant	Mosaic males	syndromic ID	Yes	PMID: 17546030 [57]; PMID: 17546031 [58]
*PQBP1*	Renpenning syndrome	Recessive	ID, microcephaly	unaffected	Yes	PMID: 15811016 [59]; PMID: 31840929 [60]
*PRPS1*	Arts syndrome	Recessive	ID, ataxia	milder phenotype	Yes	PMID: 24528855 [61]
*RLIM (RFN12)*	Tonne-Kalscheuer syndrome	Not reported	ID, GDD, Autism, congenital malformation	generally unaffected	Yes	PMID: 29728705 [62]
RAB39B	Waisman Syndrome	Recessive	ID, epilepsy, Parkinson disease	later onset Parkinson disease and unaffected	N.D.	n/a
*RPS6KA3 (RSK2)*	Coffin-Lowry syndrome	Dominant	syndromic ID, microcephaly	mild ID	Yes	PMID: 12030896 [63]
*RPS6KA3 (RSK2)*	X-Linked MR19	Dominant	mod ID	mild nonsyndromic ID	N.D.	n/a
*SLC35A2*	Congenital disorder of glycosylation, type II	Dominant	males are mosaic	Infantile epileptic encephalopathy	Yes	PMID: 24115232 [64]
*SLC6A8*	Creatine transporter deficiency	Recessive	ID, epilepsy	mild	Yes	PMID: 20528887 [65]
*SLC9A6 (NHE6)*	Christianson syndrome	X-linked	profound ID, epilepsy	ID, learning differences, ADHD, speech delay	Yes	PMID: 18342287 [66]
SLC9A7	Intellectual developmental disorder, X-linked 108	Recessive	ID	unaffected carrier females	N.D.	n/a
*SMC1A*	DEE85; Cornelia de Lange, 2	Dominant	Lethal in males; ID, limb malformations, dysmorphic	ID, midline brain defects, seizures	escape X inactivation	PMID: 30871455 [24]
SOX3	Intellectual Disability, X-linked, with isolated growth hormone deficiency	X-linked	ID, panhypopituitarism	unaffected carrier females	N.D.	n/a
STAG2	Holoprosencephaly 13, X-linked	Dominant, Recessive	early lethality, brain malformation, ID	ID, brain malformation/unaffected	N.D.	n/a
SYN1	Epilepsy, X-linked, with variable learning disabilities and behavior disorders	Dominant, Recessive	ID, epilepsy, ASD	epilepsy and ASD	N.D.	n/a
SYP	Intellectual Disability, X-linked 96	Recessive	ID, epilepsy	unaffected	N.D.	n/a
*TAF1*	XLID 33	Recessive	syndromic ID	unaffected	Yes	PMID: 26637982 [67]
*THOC2*	XLID 12/35	Recessive	mild - moderate ID	not affected	Yes	PMID: 26166480 [68]
TSPAN7	Intellectual Disability, X-linked 58	Recessive	ID	unaffected carrier females	N.D.	n/a
UBE2A	Intellectual Disability, X-linked syndromic, Nascimento-type	Recessive	ID, epilepsy	unaffected carrier females	Yes	PMID: 16909393 [69]
*UPF3B*	XLID 14	Recessive	Severe non-syndromic ID, Autism	not affected	Yes	PMID: 19238151 [70]
*USP9X*	Syndromic XLID 99	Dominant	ID, autism, maybe Lethal in males	mild or unaffected or ID, multiple congenital anomalies	escapes X inactivation	PMID: 29022598 [7]
USP27X	Intellectual Disability, X-linked 105	Recessive	ID	unaffected carrier females	N.D.	n/a
*WDR45*	NBIA5	Dominant	lethal, mosaics - affected	static encephalopathy, adult onset neurodegeneration, infantile spasms, developmental delay, ID	Yes	PMID: 23176820 [71]
*ZDHHC9*	Raymond type XLMR	X-linked	non-syndromic ID	unaffected	N.D.	n/a

## 3. MECP2

The *MECP2* gene is predominantly expressed in the brain where MeCP2 binds to methylated DNA via methyl-CpG pairs and acts as both a transcriptional repressor and an activator of gene expression [10]. MECP2 is important for prenatal neurogenesis, postnatal development of synaptic connections and function, synaptic plasticity, and adult neural function [72]. Variants involving the *MECP2* gene may result in MECP2 duplication syndrome, Rett syndrome (RTT), X-linked intellectual disability or autism spectrum disorder (ASD).

**Figure 1:**
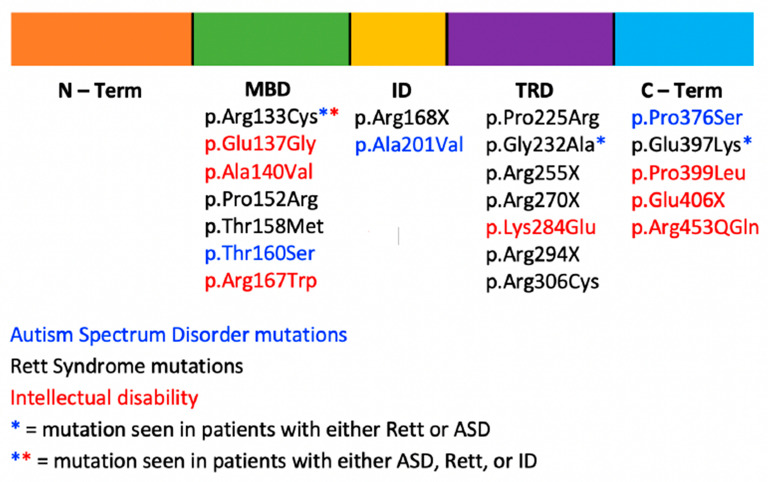
Schematic of the MECP2 protein showing phenotypic variability among pathogenic variants and their associated disorders.

Loss-of-function or missense variants in *MECP2* may results in syndromic or non-syndromic intellectual disability, Rett syndrome, or ASD without RTT (Figure 1). RTT is seen almost exclusively in females and is lethal in most males by age 2. Individuals with classic RTT generally present with normal early growth and development followed by developmental stagnation between 6 and 18 months and a period of developmental regression affecting social skills, speech, gait, and purposeful hand use between 1 and 4 years old. During the period of regression, distinct hand movements, seizures, and irregular respirations emerge. In addition to RTT, loss-of-function *MECP2* variants can also cause a non-specific X-linked intellectual disability in males and females [73]. Females often have mild intellectual disability, while males may develop mild to severe intellectual disability, including PPM-X syndrome marked by psychosis and bipolar disorder, parkinsonism, increased muscle tone, exaggerated reflexes, and abnormal enlargement of the testes [74].

The disease severity and variability of the phenotype is influenced both by the location and type of the variant as well as by genetic background and cellular environment [39]. Truncating variants in *MECP2* are associated with a more severe phenotype than missense variants, and individuals with truncations show earlier development of hand stereotypies, decreased height *z*-scores, paucity of speech [75], a higher incidence of awake respiratory dysfunction [76], and overall higher clinical severity [77].

Variant type and location do not adequately explain phenotypic variability, as individuals with the same pathogenic variant have clinical presentations varying from ASD, ID, and RTT (Figure 1), and XCI has been proposed to be an important factor in the onset and severity of RTT [78,79]. Phenotypic variation ranging from classical RTT to normal individuals with protective skewing of the X chromosome have been reported [78]. Zhang et al. described a Chinese family with Rett syndrome and X-linked intellectual disability [79]. They reported eight individuals with *MECP2* variants in six families. 

A family made up of a mother, daughter, and son had the identical *MECP2* variant c.397C > T. The daughter was diagnosed with a preserved speech variant of RTT, the son was diagnosed with X-linked mental retardation (XLMR), while the mother was healthy. XCI studies showed that the mother had skewing towards the normal allele, while the daughter had random XCI. Another mother and daughter pair were found to have the same c.397C > T *MECP2* variant. However, although they both had random XCI, the daughter was diagnosed with RTT, and the mother had learning difficulties and autistic behaviors [79]. While the variability in phenotypes between the mothers and their daughters with the same MECP2 variant may be due to the difference in the pattern of XCI, not all clinical presentations can be explained by the pattern of XCI, given that the clinical symptoms of the mother with random XCI were milder than those of her daughter with the same variant and degree of XCI. Consistent with this report, Xiol et al. found no substantial correlation between the XCI patterns in the blood and the clinical presentation of RTT. In their study of 221 RTT patients with nine recurrent *MECP2* variants or a large deletion in *MECP2*, 17 out of 174 patients had a skewed XCI pattern, and there were no consistent increases or decreases in the clinical severity score of RTT patients with a preferential inactivation of the wild-type or mutated alleles [39]. In addition, the XCI pattern in blood and cortex was different for two patients included in their study. A similar finding was reported by Bao et al., who showed no statistically significant relationship between clinical severity and pattern of XCI [80].

*MECP2* duplication results in a gain-of-function phenotype that is inherited in a recessive manner, predominantly affects males, and is characterized by severe to profound intellectual disability and limited or absent speech. Individuals with this syndrome have early-onset hypotonia and have progressive spasticity affecting the lower limbs. Additionally, 50% of affected males have epileptic seizures, and many have a predisposition to recurrent infections [81,82,83]. The X chromosome carrying the duplication is often preferentially silenced in most asymptomatic carriers [84]; however, some females have a mild phenotype, despite inactivation of the variant chromosome [85]. Symptomatic females exhibiting random XCI or skewing with preferential expression of the duplicated chromosome may present with varying severity and can exhibit learning disabilities, intellectual disability, autistic features, or psychiatric symptoms [86,87]. 

## 4. FMR1

The *FMR1* gene encodes the fragile X mental retardation protein (FMRP), an RNA-binding protein that is highly expressed in the brain and reproductive organs. FMRP regulates the translation, transport, and stability of mRNAs and plays important roles in neuronal development and synaptic plasticity [88]. *FMR1*-related disorders include fragile X syndrome (FXS), fragile X tremor/ataxia syndrome (FXTAS), and premature ovarian insufficiency (POI), and result from expansion of the trinucleotide CGG repeat in the 5′ untranslated region. The repeat is categorized into four groups based on the size of the repeat: normal alleles (5–44 repeats), intermediate alleles (45–54 repeats), premutation alleles (55–200 repeats), and full-mutation alleles (>200 repeats). The *FMR1* premutation is associated with FXTAS and POI, while the full mutation is associated with FXS. Normal alleles are typically transmitted from parent to offspring in a stable manner without any increase or decrease in repeat number. Intermediate alleles may expand into the premutation range when transmitted by the mother [89], while premutation alleles are unstable and tend to expand into a full mutation when transmitted from mother to offspring. It is estimated that about 1 in 850 males and 1 in 300 females have the premutation and 1 in 7000 males and 1 in 11,000 females have the *FMR1* full mutation [90].

### 4.1. FMR1 Full Mutation

The phenotype of the full mutation results from hypermethylation of the CGG expanded region, thus causing the loss of *FMR1* transcription and the absence of FMRP. Most males with FXS have intellectual disability, macrocephaly, facial dysmorphism, high arched palate, joint hyperlaxity, hypotonia, otitis media, pes planus, connective tissue problems, and pectus excavatum. The behavioral features typically include attention-deficit/hyperactivity disorder (ADHD), anxiety, depression, emotional lability, gaze avoidance, stereotypic movements, echolalia, and sensory processing differences. ASD is present in 50–70% of individuals with FXS [91], and epilepsy is present in 10–20% of individuals and begins between ages 4 and 10 years [92]. The physical and behavioral features seen in males with FXS are present in females with the full mutation but are typically less severe. The female phenotype is more commonly associated with learning disabilities, behavioral problems, anxiety, depression, shyness, and difficulties in establishing social interactions [93], and about half of females with FXS are diagnosed with intellectual disability [91].

The differences in phenotype among females with full *FMR1* mutations can be attributed to differences in X-chromosomal inactivation as shown in a case study of three females with the full mutation from the same family [94]. Patient III-1 had complete inactivation of the normal allele and physical traits of FXS and presented with hand-flapping, short attention span, tactile defensiveness, shyness, and poor eye contact. Less than 10% of the normal allele was inactive in patient II-1 who presented with normal intelligence, while 50% of the normal allele was inactive in patient II-2, and she presented with mild physical traits and intellectual disability [94]. Another case study described two sisters with *FMR1* full mutations with different fragile X phenotypes. One sister had severe intellectual disability and phenotypic traits like those observed in males with FXS. She had complete inactivation of the normal X chromosome, while her sister with learning disabilities had the normal X chromosome active in 70% of her cells [95]. Martorell et al. described a consanguineous Moroccan family in which the four sisters were compound heterozygote for full and pre-mutation in *FMR1*. The proband had complete inactivation of normal X chromosome and presented with autistic-like features and had severe intellectual disability, while the sisters had a random XCI pattern and had learning disabilities and emotional problems with mildly affected IQ [96]. These findings support the hypothesis that the different phenotypes in female carriers with full mutations are primarily caused by unequal X-chromosomal inactivation.

### 4.2. FMR1 Premutation

#### 4.2.1. Premature Ovarian Insufficiency (POI)

Female carriers of the fragile X premutation have an increased risk for development of premature ovarian insufficiency (POI), a condition in which women experience infertility, irregular menstruation, and menopause prior to 40 years old. Although it has been hypothesized that the development of POI in fragile X premutation carriers is due to skewed XCI, this has not been supported by published literature. Using the polymorphic androgen receptor (AR) gene assay, Spath et al. compared the inactivation patterns in female premutation carriers with POI (*n* = 37) to those of female premutation carriers without POI (*n* = 64) and women with idiopathic POI (*n* = 25). They found that the degree of skewed XCI did not differ significantly between female premutation carriers with POI, female premutation carriers without POI, and females with idiopathic POI [97]. Similarly, Rodriguez-Revenga et al., using the same methodologic approach, compared the XCI patterns from 220 control female samples, 40 female premutation carriers with POI, and 220 female premutation carriers without POI. Their results showed no significant difference in the prevalence of skewed XCI among non-POI and POI *FMR1* premutation carriers [98]. These findings were further substantiated by a study of monozygotic twins with similar sized *FMR1* premutations who had discordant phenotypes for POI and similar X-inactivation ratios [99]. The idea that the development of POI is related to CGG repeat size was proposed by Sullivan et al. In their study of 507 women, they showed that repeat sizes in the medium premutation range (80–99 repeats) were associated with the highest risk for POI, and the risk of developing POI appears to plateau, or perhaps decrease, among women with very high repeats (>or =100 repeats) [100].

#### 4.2.2. Fragile X-Associated Tremor/Ataxia Syndrome (FXTAS)

Fragile X-associated tremor/ataxia syndrome (FXTAS) is a late-onset neurodegenerative disorder that is characterized by intention tremor and gait ataxia, with more variable features of parkinsonism, short-term memory problems, and deficits in executive function. FXTAS symptoms typically affect people over the age of 50 and worsen with age. Male premutation carriers are generally more frequently and severely affected than females. XCI may play a role in facilitating the phenotypic differences between males and females with FXTAS, and it has been hypothesized that the disease severity is inversely related to the activation ratio (AR) for the normal *FMR1* allele. Although the data are limited, two case series describe sisters with similar premutation size *FMR1* alleles and found that the sisters with the lowest AR of the normal allele had the most severe FXTAS symptoms, while the sisters with the highest AR had no signs of FXTAS [101,102]. These studies suggest that the AR may play a role in the development of FXTAS and its severity in premutation carrier women. 

#### 4.2.3. Children with FMR1 Premutation

*FMR1* premutation research has primarily focused on FXTAS and POI in adults; however, children with a *FMR1* premutation are also at increased risk for several health concerns. Bailey et al. reported on 256 children with a *FMR1* premutation and their co-occurring conditions. Premutation males, when compared with the control group, were more likely to have developmental delay, attention problems, aggression, seizures, ASD, and anxiety, while premutation females were more likely to have attention problems, anxiety, depression, and developmental delay [103]. These findings are corroborated by Renda et al. [104] and Farzin et al. [105] but were in contrast to work by Myers et al. [106]. In their study of 28 children, they found no significant difference between children with and without the premutation. Although several medical conditions seem to be related to the *FMR1* premutation in children, none of the studies published to date have examined the effect of XCI, thus further study is needed.

## 5. Conclusions

Symptoms of X-linked disorders are variable among females, with some presenting the full disease phenotype, while others present with a milder phenotype or as asymptomatic carriers. Skewing of XCI provides a mechanism for the diversity of phenotypes observed in X-linked disorders, as shown by our discussion of *MECP2*- and *FMR1*-related disorders; however, it does not account for all phenotypic variability as seen in the cases of POI. The lessons learned from these disorders can be extended to other X-linked NDDs, as shown in Table 1, where the phenotypic expression of many X-linked genes is regulated by XCI. Skewed XCI may be required for survival, as it is observed in a majority of heterozygous females [107]; however, the impact of skewed XCI on phenotype is not well understood. The studies included in this review have primarily relied on blood for XCI studies. While blood is the most assessable tissue, the pattern of XCI may not correlate well with XCI in the brain. This, in addition to the small sample size are limitations of the XCI studies and indicates that XCI pattern in blood is not a useful predictor of phenotype.

Despite these challenges, targeted reactivation of genes on the inactive X chromosome could represent a therapeutic approach in heterozygous females affected by X-linked diseases, and several groups are exploring this possibility in rodent models and in-vitro cell lines [108,109]. Sex chromosomal dosage compensation is an important developmental process, and disturbing XCI could have severe consequences for females since overexpression of genes, such as *MECP2*, results in *MECP2* duplication syndrome. While more work remains to be done, these preliminary studies show promise and may lead to meaningful interventions.

## Data Availability

No new data were created or analyzed in this study. Data sharing is not applicable to this article.
